# Cushing’s syndrome: a systematic review of psychiatric and cognitive symptoms in case studies

**DOI:** 10.20945/2359-4292-2026-0029

**Published:** 2026-04-01

**Authors:** Roni Vian da Silva Lhul, Katiúscia Gomes Nunes, Tobias Skrebsky de Almeida, Mauro Antônio Czepielewski, Letícia Sanguinetti Czepielewski

**Affiliations:** 1 Universidade Federal do Rio Grande do Sul, Porto Alegre, RS, Brasil

**Keywords:** Cushing’s syndrome, psychiatric disorders, cognitive impairment, case studies, systematic review

## Abstract

**Objective:**

Cushing’s syndrome, characterized by chronic hypercortisolism, is associated with various health risks, including psychiatric symptoms and cognitive impairments. This systematic review of case studies aimed to map and categorize these symptoms.

**Materials and methods:**

We hypothesized that a broader range of psychiatric and cognitive manifestations would be observed beyond anxiety, depression, and memory impairment. The review followed PRISMA guidelines and was preregistered in PROSPERO (CRD42024433186). We conducted searches in PsycINFO, Embase, PubMed, and Scopus, identifying 273 potentially relevant studies. After screening, 66 studies were included, comprising 74 cases (81% female; mean age 35.7 years, range 13-81).

**Results:**

**Results:**

revealed that 93% of cases presented psychiatric complaints, including depression (39.2%), psychosis/schizophrenic symptoms (35.1%), suicidal ideation/attempts (20.3%), anxiety (17.5%), panic attacks (2.7%), and post-traumatic stress disorder (1.4%). Cognitive complaints were reported in 32% of cases, primarily as general cognitive complaints (18.9%), memory impairment (9.5%), and attentional deficits (5.4%). An overlap of psychiatric and cognitive symptoms was observed in 26% of cases.

**Conclusion:**

This review underscores the clinical relevance of symptoms such as mania, psychosis, and suicidal behavior in Cushing’s syndrome, which are often underreported. Individualized clinical assessment informed by these case studies is crucial for comprehensive management that extends beyond the typical focus on depression and memory. Moreover, greater awareness of the full spectrum of neuropsychiatric manifestations in hypercortisolism is needed.

## INTRODUCTION

Cushing’s syndrome (CS) is a rare neuroendocrine disorder, with an estimated prevalence of 39.1 per million (^1^), characterized by prolonged exposure to elevated cortisol levels (^2^). It may cause multiple serious health risks, including cardiovascular, metabolic, musculoskeletal, and skin disorders (^3^). Furthermore, CS presents a wide range of symptoms, with some studies reporting psychiatric manifestations, primarily anxiety and depression (^4^), and cognitive impairments, most commonly affecting memory (^5^).

Case studies can provide a broader scope of highly relevant data for studying rare diseases, as they allow exploration of less common but equally or more severe and clinically significant symptoms (^6^). A systematic review of case studies is particularly justified for rare diseases due to their low prevalence, clinical and diagnostic heterogeneity, and the scarcity of randomized controlled trials and observational studies with large enough samples to generate robust evidence. Moreover, earlier systematic reviews examining larger samples of CS have provided limited information on less common psychiatric symptoms such as mania and psychosis. Therefore, this study aimed to map psychiatric and cognitive symptoms reported in case studies of patients with CS.

## MATERIALS AND METHODS

This systematic review included case studies and series published in Portuguese, English, or Spanish that reported psychiatric and/or cognitive symptoms in patients with CS of all ages and sexes, regardless of disease stage. Studies were excluded if they were unavailable online, incomplete, or did not meet the first seven objective items of the Critical Appraisal Checklist for Case Reports (^7^). Item 8, ‘take away lessons’, was excluded due to its subjective nature and reduced relevance to methodological quality and risk of bias assessment.

**Supplementary Figure 1:**
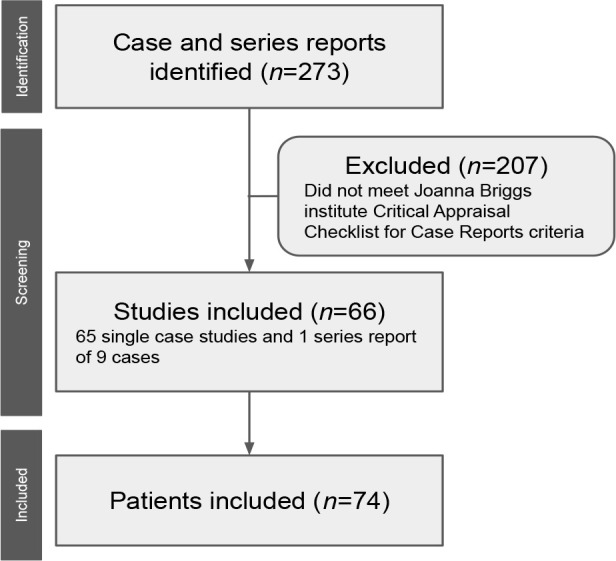
PRISMA Flow Diagram.

The methodology adhered to the Preferred Reporting Items for Systematic Reviews and Meta-Analyses (PRISMA) statement (^8^) and was preregistered under the International Prospective Register of Systematic Reviews (PROSPERO) registration no. CRD42024433186, covering studies published up to April 2024. Database searches were conducted in April 2024 using the terms “cogn*” OR “psyc*” AND “Cushing” in PsycINFO, Embase, PubMed, and Scopus. Data selection and analysis were conducted by two blinded researchers, with conflicts resolved by a third reviewer in Rayyan (^9^). Data from the selected studies were systematically tabulated in Excel. Detailed information regarding the article selection process is provided in the PRISMA Flow Diagram (see **Supplementary Figure 1**).

## RESULTS

A comprehensive search yielded 273 potentially relevant case studies. After screening based on predefined inclusion criteria, 66 case studies were included in this systematic review. Most included studies originated from the United States (n = 26), with the remainder primarily from Europe and none from Latin America. One study included a case series of nine patients, resulting in 74 individual cases analyzed. Of these, the method of cortisol measurement was explicitly detailed in 71 cases (97.2%). Regarding diagnostic imaging, 34 cases (46%) utilized magnetic resonance imaging, 31 (41.9%) used computed tomography, and 16 (21.6%) used both imaging methods.

The participants were diagnosed by an endocrinologist and exhibited psychiatric or cognitive symptoms identified during hospital evaluation. Of the 74 cases, 60 were female (81%). Ages ranged from 13 to 81 years (mean = 35.7 years; standard deviation = 15.9). Analysis of reported symptomatology revealed that a substantial majority (n = 69, 93%) presented psychiatric complaints. Cognitive complaints were reported in 24 cases (32%). Notably, 19 cases (26%) presented both psychiatric and cognitive symptoms (**Table 1**).

**Table 1:** Distribution of psychiatric, cognitive, and overlapping symptoms (psychiatric and cognitive reported in the same patient) by etiology

Etiology	Case studies (n)	Symptoms (n)		
		Psychiatric	Cognitive	Overlap
Disease	35	32	17	14
Adrenal	25	23	3	2
Ectopic	10	10	3	3
Lung	8	8	3	3
Pancreas	1	1	0	0
Undetectable source	1	1	0	0
Exogenous	4	4	0	0
All etiologies	74	69	23	19

**Figure 1:**
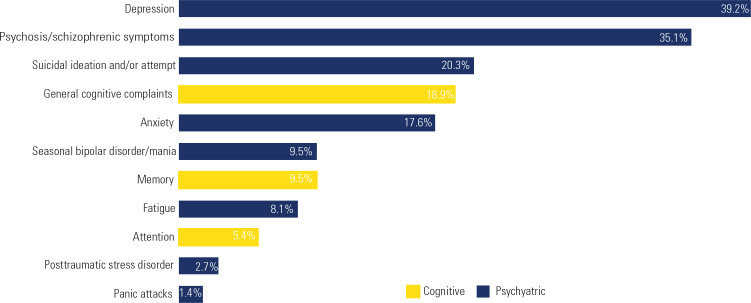
Frequency of specific psychiatric or cognitive symptoms reported in published case studies of Cushing’s syndrome.

The distribution of psychiatric symptoms (**Figure 1**) indicated that the most frequently reported were depression (n = 29, 39.2%), followed by psychosis/schizophrenic symptoms (n = 26, 35.1%), suicidal ideation and/or attempts (n = 15, 20.3%), and anxiety (n = 13, 17.5%). Less frequently reported psychiatric symptoms included seasonal bipolar disorder/mania (n = 7, 9.5%), fatigue (n = 6, 8.1%), panic attacks (n = 2, 2.7%), and post-traumatic stress disorder (n = 1, 1.4%). Regarding cognitive functioning, the most commonly reported symptoms were general cognitive complaints (n = 14, 18.9%), memory impairment (n = 7, 9.5%), and attentional deficits (n = 4, 5.4%).

## DISCUSSION

This review aimed to map and categorize the cognitive and psychiatric dysfunctions in CS through a systematic review of case studies. In addition to the high rates of anxiety and depression widely documented in the literature, we identified an increased occurrence of psychosis and suicide attempts, as well as various other less common symptoms. Most articles reporting cognitive complaints did not specify them, limiting interpretation as to whether difficulties pertained exclusively to memory or encompassed other cognitive domains. Over one-quarter of participants experienced both cognitive and psychiatric symptoms, highlighting the complex interplay between these domains. Analysis of cognitive functioning revealed a heterogeneous presentation, with varying degrees of impairment across different domains. Cognitive complaints were reported in 18.9% of cases, encompassing a range of difficulties identified through physician observation or self-report instruments. Notably, the majority of included studies relied on the Mini-Mental State Examination rather than comprehensive or objective neuropsychological assessments, with only one study employing the Trail Making Test (^10^). This lack of specificity is attributable to the absence of formal neuropsychological testing in most case reports. Consequently, the true nature of cognitive deficits in CS may be inadequately characterized by this literature. Difficulty concentrating was the most frequently reported cognitive complaint (^11^-^14^). Although less common, memory and attention deficits were also present, consistent with previous studies associating these impairments with neurocognitive dysfunction in CS (^14^,^15^).

This study underscores the need for greater awareness of the full spectrum of psychiatric manifestations in hypercortisolism. Nearly 40% of cases exhibited depressive symptoms, albeit our findings also indicate the presence of other clinically relevant symptoms such as mania, hypomania, or seasonal bipolar disorder in nearly 10% of cases. The lower frequency of manic or hypomanic episodes is consistent with reports suggesting that these symptoms are less common in hypercortisolism and often underreported (^16^). When documented, these typically appear in case studies demonstrating the emergence of mania or hypomania related to CS (^17^-^19^) or the exacerbation of existing symptoms due to hypercortisolism (^20^). In contrast, psychosis was reported in 35% of cases, even though it is less commonly associated with CS. This discrepancy is likely due to the bias inherent in case studies, in which such conditions are more frequently documented because of their rarity and severity (^21^-^24^).

Panic attacks are less frequently reported in the literature (^25^), despite some authors indicating them as the third most prevalent psychiatric disorder in hypercortisolism (^26^). In this review, both identified cases of panic attacks also involved psychosis, suggesting a possible correlation between these symptoms in CS (^25^,^27^). Fatigue also appears to be common in CS, with studies citing rates of up to 42% (^28^), while our findings up to 8.1%, suggesting underreporting in case studies, perhaps due to lower risk of mortality. Only one case had post-traumatic stress disorder symptoms, comorbid with psychosis (^11^); previous sample studies have suggested a correlation between CS and persistent post-traumatic stress disorder lasting over one year (^29^). Finally, the presence of suicidal ideation and/or attempts in 20.3% of cases shows the substantial clinical impact of hypercortisolism on mental health. Even after remission, patients may continue to experience significant psychiatric outcomes and risk of suicide (^30^). Given these findings, the scarcity of observational studies specifically investigating suicidal ideation in CS is concerning.

This study should be interpreted in light of several limitations. First, the results should be interpreted cautiously as they derive from highly heterogeneous case studies. A key limitation is the scope of the search strategy: we focused on major international databases and did not include other relevant databases (e.g., Psicodoc, Biblioteca Virtual em Saúde, and Web of Science), the grey literature, and studies published in other languages. Furthermore, most studies represented WEIRD (Western, Educated, Industrialized, Rich, and Democratic) populations, thereby not adequately represent diverse populations worldwide. Despite these limitations, the results are consistent with the literature that provides robust evidence for the relationship between cortisol and major depressive and anxiety disorders, panic, post-traumatic stress, and cognitive complaints. Future research would benefit from standardized neuropsychological batteries to go beyond general complaints, enabling a more precise characterization of cognitive impact in CS.

## CONCLUSION

The findings of this systematic review of CS case studies highlight the importance of closely monitoring symptoms such as depression, anxiety, and cognitive complaints, including memory problems. Importantly, our study emphasized the presence of additional symptoms, including mania, psychosis, panic attacks, post-traumatic stress disorder, and suicide attempts, which are often linked to CS but frequently underreported in observational studies. Comprehensive psychological and psychiatric evaluation is crucial for accurate mental health diagnosis and appropriate, individualized intervention. This approach is essential to providing comprehensive, personalized care that may enhance these patients’ quality of life.

## Data Availability

the datasets used and/or analyzed during the current study available from the corresponding author on reasonable request.
